# Moderately Reducing Nitrogen Application Ameliorates Salt-Induced Growth and Physiological Damage on Forage Bermudagrass

**DOI:** 10.3389/fpls.2022.896358

**Published:** 2022-04-29

**Authors:** An Shao, Hongli Wang, Xiao Xu, Xiaoning Li, Erick Amombo, Jinmin Fu

**Affiliations:** Coastal Salinity Tolerant Grass Engineering and Technology Research Center, Ludong University, Yantai, China

**Keywords:** forage bermudagrass, salt stress, nitrogen application, physiological response, forage quality

## Abstract

Nitrogen (N) application is one of the most effective methods to alleviate salt-induced damage on plants. Forage bermudagrass has higher utilization potential on saline soil, but whether its N requirement changed under high salt stress has not been studied. Through examining plant growth-related traits, salt-stress-responsive physiological traits, photosynthesis, N metabolism, and forage quality supplied with different N concentrations under high salt stress (200 mM NaCl), we noticed that the optimum N requirement of forage bermudagrass reduced. When supplied with 10 mM N under higher salt stress, plants had a similar biomass, turf color, and chlorophyll content with plants supplied with 15 mM N, accompanied by a lower firing rate and Na^+^ content of leaves. The N content, crude protein, crude fat content, the expression of *AMTs* (ammonium transporters), *NR* (nitrate reductase), *GS* (glutamine synthetase), and *GOGAT* (glutamate synthetase), the chlorophyll fluorescence curve, and parameters of leaves (e.g., PI_ABS_; PI_CS_; ABS/RC; TRo/RC; ETo/RC) all peaked under 10 mM N under high salt stress instead of 15 mM N. Through exploring the proper N application under higher salt stress and its alleviation mechanisms, our results indicated that moderate reduction in N application under high salt level had a maximum promotion effect on the salt tolerance of forage bermudagrass without growth or forage quality inhibition. These response mechanisms obtained can provide a useful reference for N application in moderation rather than in excess on forage bermudagrass, especially in higher salinity areas.

## Introduction

Bermudagrass [*Cynodon dactylon* (L.) Pers.] is one of the most widely used grass species as forage and turfgrass in warm climatic regions. Bermudagrass was also considered to be a salt-tolerant grass species (Maas and Hoffman, 1977; Chen and Liu, 2012). However, the growth, forage quality, and turf quality of bermudagrass could be seriously inhibited by salt stress (Marcum and Pessarakli, 2006), greatly limiting its wide application in salinity areas. Salt stress generally disrupts the osmotic and ionic equilibrium in bermudagrass cells, which would lead to ionic toxicity, osmotic stress, and secondary stresses (Ahanger et al., 2019; Maksup et al., 2020; Amin et al., 2021), including nutritional imbalance, inhibition of photosynthesis, and membrane disorganization (Munns and Tester, 2008). Like other plants, bermudagrass has established corresponding response mechanism in acclimation to external salt stress to reduce their damage (Deinlein et al., 2014; Zhao et al., 2020), such as the induction of antioxidant enzymes (Baby and Jini, 2010; Ahmad et al., 2019), ion transport and compartmentation, compatible solutes synthesis, and accumulation (Zhao et al., 2020).

Nitrogen (N) fertilizer plays a critical role in alleviating salt-induced damage in plants (Mansour, 2000; Villa et al., 2003; Esmaili et al., 2005; Jia et al., 2017). As the most important macro-nutrient of plants, N is an important constituent of the nucleic acids, proteins, chlorophyll, and many other N metabolites (Kishorekumar et al., 2020). N also mediates the utilization of potassium, phosphorus, and other elements and serves as an important component of proteins involved in a series of metabolic processes to coordinate plant growth and development (Zubillaga et al., 2002). Two primary inorganic N sources ammonium (NH_4_^+^) and nitrate (NO_3_^–^) from soil can be directly absorbed by roots and are transported to plants by their respective transporters ammonium transporters (AMTs) and nitrate transporters (NRT) (Giagnoni et al., 2016; Dechorgnat et al., 2019). The NH_4_^+^ absorbed must be transformed into NO_3_^–^ under the catalyst of nitrate reductase (NR). Then, NO_3_^–^ can be further assimilated and converted into amino acid through glutamine synthetase (GS) and glutamate synthase (GOGAT) metabolic pathway, which was considered to be the main pathway for N assimilation in plants (Xu et al., 2012).

However, reduced N uptake of plants always occurs due to the high Na^+^ and Cl^–^ content in the saline soil (Akram et al., 2011). Salinity results in significantly decreased activities of NR, GS, and GOGAT in many plants species, such as maize and rice (Khan and Srivastava, 1998; Wang et al., 2012), and further inhibits the N use efficiency in plants (Murtaza et al., 2013; Singh et al., 2016; Teh et al., 2016). Moreover, several N-containing compounds could be induced by salt stress and contributed to salt tolerance of plants (Hoai et al., 2003; Dluzniewska et al., 2007; Sudmalis et al., 2018) through participating in osmotic adjustment, the promotion of photosynthetic capacity, and mitigation of oxidative stress caused by excessive ROS (Homaee et al., 2002; Song et al., 2006). Therefore, N application is considered to be one of the most effective methods of improving plant growth in salinity regions where N content is lower than that in non-salinity land.

Related research also showed that, rather than N alone, plant growth was significantly affected by the interaction between soil salinity and N. Some reports showed that the alleviation effects of N fertilizer supplied might associate with its concentrations supplied. For instance, low-to-moderate N application could mitigate the adverse effects from salt stress, while excessive N could aggravate the negative effects of salt stress on cotton (Chen et al., 2010). Moreover, excessive application or inefficient use of N fertilizer will lead to secondary salinization, which in turn adversely inhibits plant growth (Soussi et al., 1998; Chen et al., 2010; Lacerda et al., 2016). In ryegrass, moderately reducing N application has the maximum alleviation effect, especially under mild salt stress (Shao et al., 2020). To sum up, the N requirement of plants might be changed (demand goes up or down) under a certain level of salt stress (higher and lower). However, there were no consistent regular changes in the N requirement under different salt concentrations or among different species.

Although bermudagrass has higher utilization potential on saline soil, excessive N application on higher levels of saline soil that cannot be fully utilized by bermudagrass might contribute to N leaching and lead to groundwater pollution. To this end, we attempt to reveal the physiological response mechanism of bermudagrass to salt stress after moderate N application by synthetically analyzing the growth rate, physiological indicators, quality-related traits, and the metabolism of N under control and salt stress treatment with different N supplied levels. The response mechanism obtained will be important for the agricultural practice to guide rational N fertilization application on forage bermudagrass cultures to further improve their salt stress resistance, maintain their growth and quality, and promote their utilization, especially under severe salt stress.

## Materials and Methods

### Plant Materials and Growth Conditions

A common tetraploid bermudagrass cultivar “Wrangle” was used in this experiment. The experimental materials used for treatment were generated by asexual propagation using the cuttings at the top of each branch from the original bermudagrass plants cultured in the greenhouse. The cuttings were planted in a solid medium (sand applied with Hoagland solution) for 1 week, and then, the same number of uniform stolons (about 20 stolons per pot) in each pot was retained in the solid medium. After 1 month, the plants of each pot were then removed from the solid medium and washed clean with water. Before the treatments were initiated, the plants were cultured in Hoagland solution to acclimate for 1 week. Subsequently, the plants were cut to the same height and transferred into different treatments in a hydroponic culture using a modified Hoagland solution. Each treatment contains four replicates. The treatments used NH_4_NO_3_ as an N source. The other components included 0.2 mM KH_2_PO_4,_ 1 mM MgSO_4,_ 1.5 mM KCl, 2.5 mM CaCl_2_, 1 × 10^–3^ mM H_3_BO_3_, 5 × 10^–5^ mM (NH_4_)_6_Mo_7_O_24_, 5 × 10^–4^ mM CuSO_4_, 1 × 10^–3^ mM ZnSO_4_, 1 × 10^–3^ mM MnSO_4_, and 0.1 mM Fe(III)-EDTA. The hydroponic culture media were processed in a growth chamber under the following conditions: 22/18°C (day/night), 60% relative humidity, 450 μmol m^–2^ s^–1^ photons, and a 16-h day/8-h night cycle. The culture solution was refreshed every 2 days.

### Experimental Design

The salt treatment condition of bermudagrass was determined based on previous studies (Dudeck et al., 1983; Adavi et al., 2006) through a simple and fast short-term salt treatment experiment (Marcum and Pessarakli, 2006; Chen et al., 2008). A pre-experiment (experiment 1) was conducted to observe the salt-induced damage of bermudagrass supplied with different N levels (2, 5, 10, and 15 mM) grown under different NaCl levels (a single step—up to 0, 50, 100, and 200 mM, respectively). After 1 month of treatment, the plant growth rate and leaf Na^+^ content were detected. According to the results of pre-experiment, 2 mM (deficiency), 10 mM (moderate), and 15 mM (excessive) N levels were chosen to explore the underlying alleviation mechanism of the reduction in N demand under high salt stress (a single step—up to 200 mM NaCl) in the subsequent experiment (experiment 2). After 1-month treatment of experiment 2, the plant growth-related trait (plant height, biomass, turf color, and leaf firing rate), salt-stress-responsive physiological traits (ion content and antioxidant enzyme activities), nitrogen content, chlorophyll content, chlorophyll a fluorescence transient, and quality-related traits of samples were measured and the gene expression involved in N metabolism was analyzed.

### Measurements

#### Plant Growth-Related Trait

Before treatment of experiments 1 and 2, the plant height and biomass of each sample were recorded. After treatment for 1 month, the plant height and the relative increment of biomass were determined according to the method described by Hu et al. (2013). The leaf color (based on a scale of 1–9, with 9 being best) and leaf firing rate were valued based on visual inspection (Fu and Huang, 2001).

#### Ion Content

After treatment, the plant samples were dried at 130°C for half an hour and then placed at 75°C for 3 days to further determine ion contents. 0.1 g dried sample was finely ground and then digested with 10 mL sulfuric acid using graphite digestion apparatus (SH220N; Hanon, Jinan, Shandong, China) with a temperature of 420°C for 2 h. After digestion, the supernatant was diluted 5–10 times. Na^+^ and K^+^ contents of each sample were measured by a flame spectrophotometer (F-500; Shanghai, China) based on the method as previously described (Fu and Huang, 2001).

#### Antioxidant Enzyme Activity

Fully expanded fresh leaves (0.3 g) were ground into powder with liquid nitrogen. Ice-cold phosphate buffer (4 mL; 50 mM, pH 7.8) was added to the powder. All the samples were centrifuged at 12,000 rpm for 20 min at 4°C. The supernatant was collected to measure the activity of antioxidant enzyme based on the method as previously described (Fu and Huang, 2001).

#### Nitrogen Content and Quality-Related Traits

After the samples were dried, green leaves, old leaves, stems, and roots were separated and ground into powder using a grinder (DFT40; Jiuping, Wuxi, Jiangsu, China). Then, the samples were digested with 10 mL sulfuric acid using graphite digestion apparatus under the temperature of 420°C for 2 h. The nitrogen content and crude protein content of samples were determined by the Kjeldahl apparatus. The Soxhlet extraction method was used to determine the crude fat content. The crude fiber content was measured according to a protocol by the International Standard Organization (2000) intermediate filtration method. The weighed samples were boiled by adding H_2_SO_4_ followed by NaOH. The extracted residue was dried at 130°C for 2 h (KSL-1200X; KeJing, Hefei, Anhui, China). Then, the dried sample was weighed and put in a furnace (500°C for 3 h). Finally, the crude fiber content of each sample was weighed. The crude ash content was determined by the high-temperature burning method. Samples were weighed and put into the muffle furnace. The temperature of the muffle furnace was set to 580°C for 5 h, and then, the crude ash of each sample was weighed. The quality-related traits were determined according to the AOAC method.

#### Chlorophyll Content and Chlorophyll a Fluorescence Transient

After treatment, leaves (fresh weight: 0.1 g) were chopped and placed into centrifuge tubes containing 4 mL dimethyl sulfoxide for 3 days. One milliliter of extract and 2 mL of dimethyl sulfoxide were mixed. The UV absorbance of each mixture was measured at 663 and 645 nm wavelengths by a UV spectrophotometer (UV-1700; Meixi, Shanghai, China). Chlorophyll fluorescence transient (OJIP curve) was determined using a pulse-amplitude modulation fluorimeter (PAM 2500, Heinz Walz GmbH). Before measurement, the plants were pre-processed in the dark for half an hour and then exposed to 3,000 μmol photons m^–2^ s^–1^ red light condition. Each treatment was repeated three times. The chlorophyll fluorescence parameters were further determined according to the calculation method described previously (Yusuf et al., 2010).

### Gene Expression Analysis

Fresh leaves of each sample were quickly ground into powder with liquid nitrogen. RNA was extracted using RNA pure Plant Kit (Tiangen Biotech, Beijing, China) and then reverse-transcribed using ReverTra Ace qPCR RT Kit (Applied Biosystems, Foster City, CA). Quantitative real-time RT-PCR analysis was performed using SYBR Green real-time PCR master mix (YeSen, Shanghai, China) and ABI real-time PCR system (Applied Biosystems, Foster City, CA) as described before (Hu et al., 2013). The relative expression level of each gene was determined according to the 2^–Δ*Ct*^ method (Vandesompele et al., 2002). The technical requirement of RT-qPCR fitted MIQE Guidelines (Bustin et al., 2009). Specific primers of selected genes are listed in Supplementary Table 6.

### Statistical Analysis

Two-way analysis of variance (ANOVA) was used with “N” and “Salt” as two main factors and “N*Salt” as one interaction term. The significance level was set at *P* < 0.05 as a threshold to analyze the significant affection of individual factors and their interactions with detected variables in this study. Histogram results were expressed as mean ± *SD* of four replicates. Tukey’s test was used to evaluate the effects of N and Salt application.

## Results

### Plant Growth and Na^+^ Content Under Different Nitrogen and Salt Levels

**FIGURE 1:**
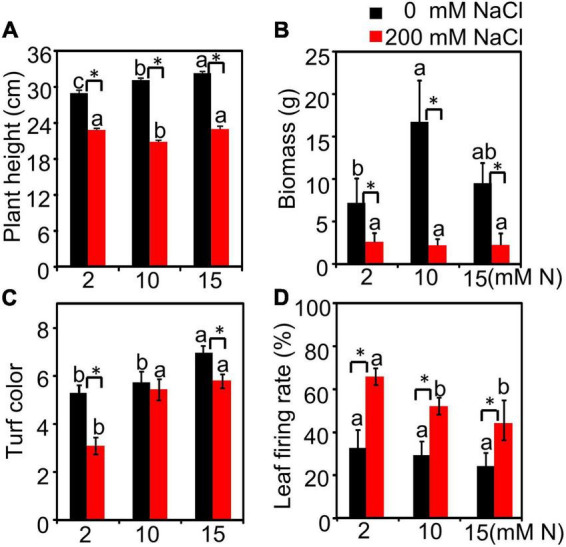
Morphological parameters of bermudagrass grown under different treatments. The plants were cut to the same height and then transferred into different nitrogen levels (2, 10, and 15 mM) under NaCl (0 and 200 mM) treatment in hydroponic culture. After treatment, the plant height **(A)**, biomass **(B)**, leaf color **(C)**, and firing rate **(D)** were measured. Different letters above the columns indicate statistically significant differences at *P* < 0.05 under different N levels with the same NaCl level by Tukey’s test. * Shows significant differences (*P* < 0.05) under 200 mM NaCl treatment and 0 mM NaCl condition with the same N level by Tukey’s test.

We first made a pre-experiment to observe the salt-induced damage of bermudagrass supplied with different N levels (2, 5, 10, and 15 mM) grown under different NaCl levels (0, 50, 100, and 200 mM) (Supplementary Figure 1). Under control (0 mM NaCl) and low salt conditions (50 and 100 mM NaCl), the plant height peaked at 15 mM N (Supplementary Figure 1A). After a higher NaCl exposure (200 mM NaCl), the plant height showed no significant difference among different N levels (Supplementary Figures 1A,C). Moreover, plants grown under 10 mM N had the lowest leaf Na^+^ content under 200 mM NaCl treatment (Figure 1B). We then chose 2 mM N (deficiency), 10 mM N (moderate), and 15 mM N (excessive) and did a separate experiment to focus on the alleviation mechanism of moderate N application at high salt levels (200 mM NaCl). The results showed a similar tendency to the pre-experiment. Without NaCl treatment, the plant height increased with the increase in N concentration and peaked under 15 mM N, while the biomass peaked under 10 mM N and then declined under 15 mM N. When exposed to 200 mM NaCl, the biomass of plants had no significant alteration among different N levels (Figure 1B). Besides, both N and salt significantly affected plant height (Supplementary Table 1 and Figures 1A,B). Plant biomass was significantly affected by salt, but not by N (Supplementary Table 1 and Figure 1B). The interactive effect of N by salt was significant for both plant height and biomass (Supplementary Table 1). Moreover, the plants supplied with 10 mM N under salt stress had a similar turf color (Figure 1C) and a lower leaf firing rate (Figure 1D) compared with the plants supplied with 15 mM N.

### Na^+^ Content Affected by Nitrogen Levels Under High Salt Stress

**FIGURE 2:**
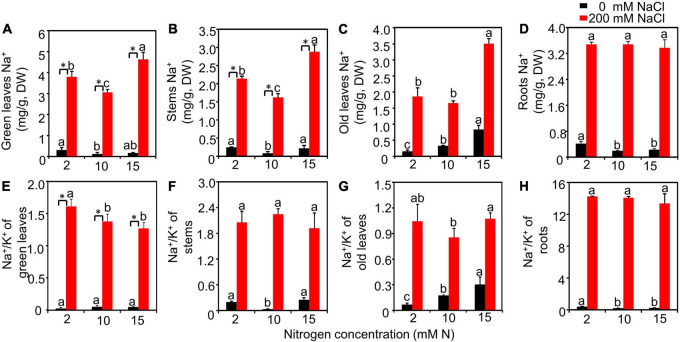
The Na^+^ content and Na^+^/K^+^ of the bermudagrass under different treatments. Na^+^ content of green leaves **(A)**, stems **(B)**, old leaves **(C)**, and roots **(D)** and Na^+^/K^+^ of green leaves **(E)**, stems **(F)**, old leaves **(G)**, and roots **(H)** of plants grown with different nitrogen concentrations exposed to different NaCl levels, respectively. Different letters above the columns indicate statistically significant differences at *P* < 0.05 under different N levels with the same NaCl level by Tukey’s test. * Shows significant differences (*P* < 0.05) under 200 mM NaCl treatment and 0 mM NaCl condition with the same N level by Tukey’s test.

The Na^+^ and Na^+^/K^+^ of different tissues detected from different treatments were further measured. The results showed that salt had a significant effect on Na^+^ and Na^+^/K^+^ of all detected tissues, while N had no significant effect on roots Na^+^ and Na^+^/K^+^ of green leaves and roots. The interactive effect of N by salt was significant for Na^+^ of green leaves, stems, old leaves, and roots and the Na^+^/K^+^ of green leaves, old leaves, and roots (Supplementary Table 2). Under the high salt condition, the Na^+^ contents of green leaves and stems had the lowest values when plants were grown with 10 mM N (Figures 2A,B). Moreover, the Na^+^ content of old leaves grown under 10 mM N had a significantly lower value compared with that grown under 15 mM N (Figure 2C). However, the content of Na^+^ of roots showed no significant difference among different N levels (Figure 2D). When grown with 10 or 15 mM N, the Na^+^/K^+^ of green leaves significantly declined compared with 2 mM N under salt stress. However, the Na^+^/K^+^ of plants grown with 10 or 15 mM N showed no significant difference (Figure 2E). Moreover, in the stems, the value of Na^+^/K^+^ showed no significant difference among different N levels under high salt stress (Figure 2F). When grown under 10 mM N, the Na^+^/K^+^ of old leaves had a significantly lower value compared with that grown under 15 mM N (Figure 2G), while the Na^+^/K^+^ of roots had no significant difference among N levels (Figure 2H).

### Antioxidant Enzyme Activities Affected by Nitrogen Levels Under High Salt Stress

**FIGURE 3:**
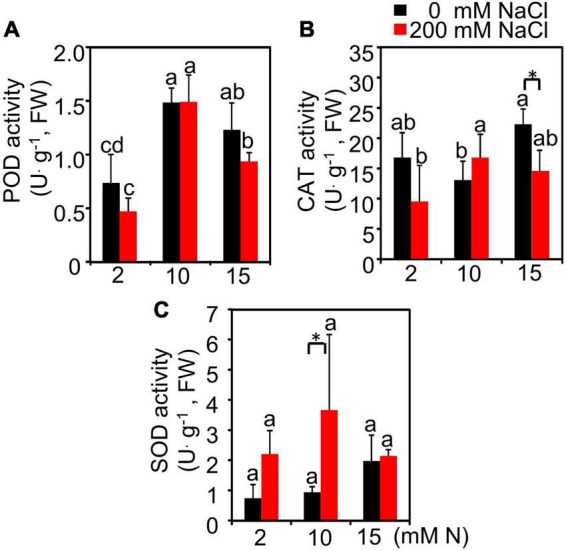
Effect of nitrogen application on the antioxidant enzyme activities of bermudagrass under control and salt treatment. POD activity **(A)**, CAT activity **(B)**, and SOD activity **(C)** in the leaves of plants grown with different nitrogen concentrations (2, 10, and 15 mM N) exposed to control (0 mM NaCl) and salt treatment (200 mM NaCl), respectively. Different letters above the columns indicate statistically significant differences at *P* < 0.05 under different N levels with the same NaCl level by Tukey’s test. * Shows significant differences (*P* < 0.05) under 200 mM NaCl treatment and 0 mM NaCl condition with the same N level by Tukey’s test.

The antioxidant enzyme activities in the leaves of plants grown under different treatments were also determined. The results showed that, with the increase in N application level, the peroxidase (POD) enzyme activity showed an upward trend and peaked at 10 mM N level followed by a downward trend under both control and salt conditions (Figure 3A). Under control conditions, the catalase (CAT) enzyme activity had a higher value when the plants were supplied with 15 mM N compared with 10 mM N (Figure 3B). After 200 mM NaCl exposure, the CAT and superoxide dismutase (SOD) enzyme activity of leaves increased slightly when the plants were grown under 10 mM N condition compared with 2 and 15 mM N conditions, but the difference was not significant (Figures 3B,C).

### Nitrogen and Protein Content Affected by Nitrogen Levels Under High Salt Stress

**FIGURE 4:**
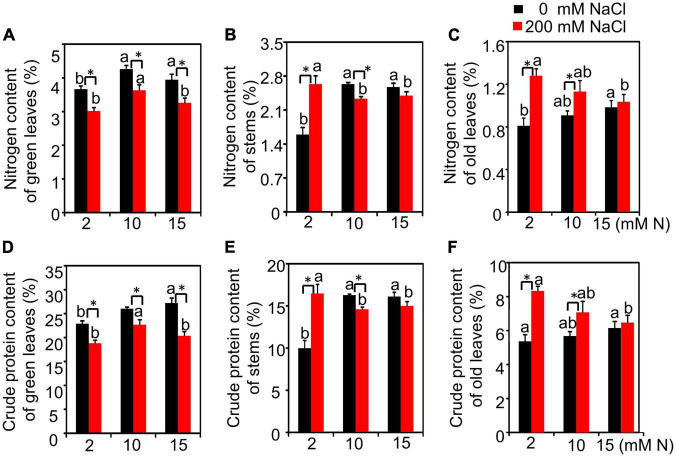
The nitrogen content and crude protein content of bermudagrass under different treatments. Nitrogen content of green leaves **(A)**, stems **(B)**, and old leaves **(C)**. Crude protein of green leaves **(D)**, stems **(E)**, and old leaves **(F)**. Different letters above the columns indicate statistically significant differences at *P* < 0.05 under different N levels with the same NaCl level by Tukey’s test. * Shows significant differences (*P* < 0.05) under 200 mM NaCl treatment and 0 mM NaCl condition with the same N level by Tukey’s test.

We then measured the N content and crude protein of green leaves and the residual N and crude protein content in old leaves and stems from different treatments. The results showed that N had a significant effect on N content or crude protein of green leaves and stems but had no significant effect on N content or crude protein content of old leaves. The effect of salt and the interactive effect of N by salt were significant for these variables measured (Supplementary Table 3). Without salt treatment, the N content of green leaves increased with the increase in N concentration and peaked under 15 mM N. After 200 mM NaCl exposure, the N content of green leaves peaked under 10 mM N and then declined under 15 mM N (Figure 4A). Without NaCl treatment, the residual N content in stems of plants grown with 2 mM N had a significantly lower value than those grown with 10 or 15 mM N. When 200 mM NaCl was added, the residual N content in stems of plants had the lowest value under 10 mM N (Figure 4B). Under high salt conditions, the residual N content in old leaves significantly increased under all three N levels. In addition, the residual N content in the stems of plants grown with 2 mM N was lower than those grown with 10 or 15 mM N (Figure 4C). The crude protein content of green leaves grown under control conditions peaked under 15 mM N, while it peaked under 10 mM N when the plants were grown under high salt conditions. This trend was consistent with that of the N content of green leaves (Figure 4D). The crude protein of stems and old leaves showed a significant increase under a high level of N application (15 or 10 mM) compared with a low level of N application (2 mM). After 200 mM NaCl exposure, although the crude protein of old leaves decreased when plants were grown under higher N levels (10 or 15 mM), the residual crude protein of stems has no significant difference among all three N levels (Figures 4E,F).

### Nitrogen Metabolism-Related Gene Expression Affected by Nitrogen Levels Under High Salt Stress

**FIGURE 5:**
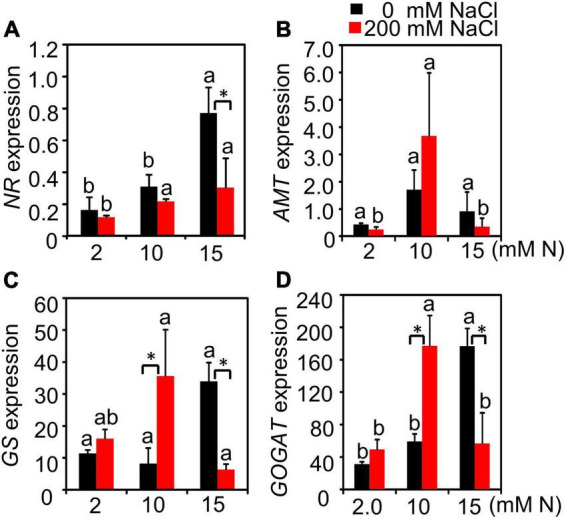
Relative expression level of N metabolism-related genes of bermudagrass under different treatments. The expression level of *NR* **(A)**, *AMT* **(B)**, *GS* **(C)**, and *GOGAT* **(D)** in the leaves of bermudagrass grown under different nitrogen concentrations exposed to different NaCl levels (0 and 200 mM NaCl), respectively. Different letters above the columns indicate statistically significant differences at *P* < 0.05 under different N levels with the same NaCl level by Tukey’s test. * Shows significant differences (*P* < 0.05) under 200 mM NaCl treatment and 0 mM NaCl condition with the same N level by Tukey’s test.

The N content and crude protein of green leaves and the residual N and crude protein content in old leaves and stems from different treatments were further determined. N had a significant effect on *NR*, *AMT*, and *GOGAT* expression, while salt only had a significant effect on *NR* expression. The interactive effect of N by salt was significant for the expression of all genes detected (Supplementary Table 4). Without NaCl treatment, the expression of *NR* increased with the increase in N concentration and peaked at 15 mM N. After 200 mM NaCl exposure, although *NR* expression was inhibited by salt, there was no significant difference among different N concentrations (Figure 5A). *AMT* gene expression showed no significant difference among plants grown with different N concentrations under control conditions. Under high salt stress, the expression level of *AMT* showed an increased trend compared with the control condition when plants were grown with 10 mM N and the expression level was significantly higher than the other two N concentrations. On the contrary, the expression level of *AMT* decreased under 2 or 15 mM N (Figure 5B) after 200 mM NaCl exposure. Under control conditions, the expression of *GS* and *GOGAT* both peaked under 15 mM N. Salt stress significantly induced the expression of GS when supplied with 10 mM N, while the expression of *GS* was reduced significantly by salt when the plants were supplied with 15 mM N (Figure 5C). Moreover, under high salt stress conditions, the expression of *GS* and *GOGAT* both peaked under 10 mM N and then significantly declined under 15 mM N (Figures 5C,D).

### Chlorophyll Fluorescence Affected by Nitrogen Levels Under High Salt Stress

**FIGURE 6:**
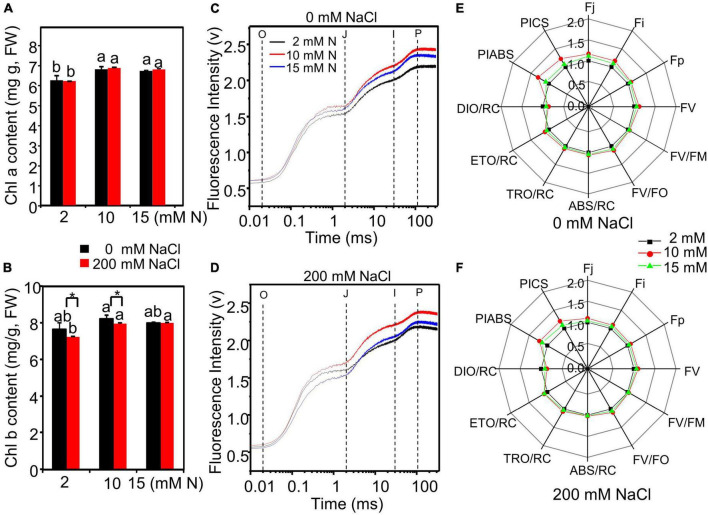
Chlorophyll fluorescence in the leaves of bermudagrass grown with different nitrogen levels under control and salt treatment. Chlorophyll a content **(A)** and total chlorophyll content **(B)** and OJIP curve in the leaves of bermudagrass grown with different nitrogen levels (2, 10, and 15 mM) under 0 mM **(C)** and 200 mM NaCl **(D)** concentrations, respectively (O: at 0.2 ms; J: at 2 ms; I: at 30 ms; P: at maximum fluorescence position). “Radar plots” of picked parameters characterizing the influence of nitrogen concentration on PS II of bermudagrass grown under 0 mM **(E)** and 200 mM NaCl **(F)**, respectively. The parameters of plants grown under 2 mM N were set as control. Control = 1. * Shows significant differences (*P* < 0.05) under 200 mM NaCl treatment and 0 mM NaCl condition with the same N level by Tukey’s test.

The total chlorophyll content of leaves grown with different treatments was measured. Both N and salt significantly affected total chlorophyll content. The interactive effect of N by salt was not significant on total chlorophyll content (Supplementary Table 1). NaCl exposure did not significantly affect chlorophyll content as compared to the control condition (Figures 6A,B). However, the chlorophyll a and total chlorophyll content of leaves reached the highest level when the plants were grown under 10 mM N and then decreased slightly under 15 mM N with or without NaCl treatment (Figures 6A,B).

Then, the impact of N levels on the photochemistry of photosystem II (PS II) of NaCl-treated bermudagrass was determined through chlorophyll a fluorescence transient–JIP test. Under control or salt condition, the fluorescence of the I and P phases of leaves grown with 10 mM N was stronger than those grown with 15 mM N, respectively (Figure 6). In particular, the OJIP curve was much higher when plants were exposed to salt under 10 mM N compared with plants grown with 15 mM N under control conditions (Figures 6C,D). The chlorophyll fluorescence parameters were further determined. Under control and salt conditions, the PI_ABS_ and PI_CS_ values, representing the overall activity of PSII, peaked under 10 mM N (Figures 6E,F and Supplementary Figures 2A,B). The absorption flux per RC (QA-reducing PSII reaction center) (ABS/RC) (Figure 6 and Supplementary Figure 2C), trapped energy flux per RC (TRo/RC) (Figure 6 and Supplementary Figure 2D), and electron transport flux per RC (ETo/RC) (Figure 6 and Supplementary Figure 2E) of plants grown with 10 mM N had a higher level or were similar to the levels of those grown with 2 or 15 mM N under control and salt conditions. On the contrary, dissipated energy flux per RC (DIo/RC) had a lower level under 10 mM N compared with other N levels under control and salt conditions (Figures 6E,F and Supplementary Figure 2F).

### Quality-Related Traits Affected by Nitrogen Levels Under High Salt Stress

**FIGURE 7:**
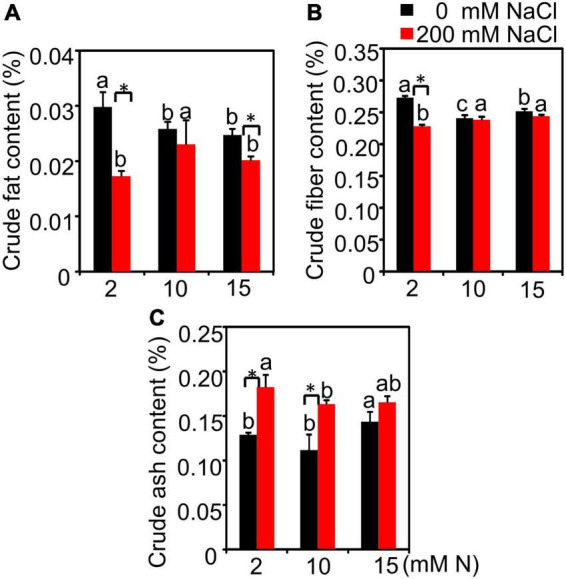
The quality-related traits of bermudagrass under different treatments. Crude fat content **(A)**, crude fiber content **(B)**, and crude ash content **(C)** in the leaves of plants grown with different nitrogen concentrations (2, 10, and 15 mM N) exposed to different NaCl levels (0 and 200 mM NaCl), respectively. Different letters above the columns indicate statistically significant differences at *P* < 0.05 under different N levels with the same NaCl level by Tukey’s test. * Shows significant differences (*P* < 0.05) under 200 mM NaCl treatment and 0 mM NaCl condition with the same N level by Tukey’s test.

Salt had a significant effect on crude fat, crude fiber, and crude ash content, while N had a significant effect on crude fiber and crude ash content, but not crude fat. The interactive effect of N by salt was significant for all quality-related traits detected (Supplementary Table 5). Without salt treatment, the increased N application significantly decreased the crude fat content. The crude fat content had the highest value when plants were grown under 2 mM N compared with 10 or 15 mM N. After 200 mM NaCl exposure, the crude fat content significantly declined under 2 mM N, while the crude fat content was not obviously affected under 10 or 15 mM N (Figure 7A). Under 0 mM NaCl condition, the crude fiber content had the lowest value under 10 mM N. When exposed to NaCl, the crude fiber content of plants grown under 10 mM N was higher than those grown under 2 mM N. However, the value showed no significant difference with plants grown under 15 mM N (Figure 7B). Moreover, the ash content had the lowest value when plants were grown with 10 mM N under both control and salt treatment conditions (Figure 7C).

## Discussion

Salt-induced inhibition, such as ion toxicity, osmotic effects, and nutrient imbalance, can be alleviated by the moderate use of N fertilizer (Chen et al., 2010; Fan et al., 2013; Duan and Chang, 2017). In forage, such as pasture grass, moderate N application has a positive effect on plant growth and yield production (Irshad et al., 2009; Shao et al., 2020). Due to possible reduction in the N requirement under higher salt stress in forage bermudagrass (10 mM N under high salt stress compared with 15 mM N under control condition) according to the plant growth rate, Na^+^ content, and antioxidant enzyme activity alteration, we mainly focused on exploring the underlying mitigation mechanism after moderate N application under high salt stress.

Salt stress can affect N uptake in plants (Murtaza et al., 2013). The ammonium N (NH_4_^+^) transported by AMT can be directly assimilated by plants (Giagnoni et al., 2016). In forage bermudagrass, after a high level of NaCl exposure, the expression of *AMT* had the highest value when plants were grown with 10 mM N compared with other N levels (Figure 5B). Previous studies reported that *AMT* gene expression was downregulated by salt in *Brassica juncea* (Goel and Singh, 2015), while most *AMT*s were upregulated in the moderate salt-treated roots of *Populus simonii* (Zhang et al., 2014). The different expression alteration of *AMTs* in different species might be due to the differential interaction of N and salt levels. In forage bermudagrass, N application could promote the expression of *AMT* within a certain range, suggesting that excessive NH_4_^+^ application had no positive effect on N absorption by upregulating the *AMT* expression under high salt conditions. Moreover, moderate N application without excess under high salt stress could help forage bermudagrass maintain or promote the expression level of N assimilation-related enzymes, such as NR, GS, and GOGAT, which can be affected by salt (Wang et al., 2012; Parul et al., 2015; Figure 5). Moreover, the green leaves N content also had the highest value when plants were supplied with 10 mM N, but not 15 mM N (Figures 4A,B) after NaCl exposure, which was consistent with the trend of N metabolism-related gene expression. Besides, the highest value of green leaves N content and lowest value of stems N content supplied with 10 mM N suggested that the moderate N application might promote the N accumulation in green leaves and reduce the residual N in stems or old leaves, which played a critical role in the rational distribution of N under salt stress.

N is a structural element of chlorophyll, affecting the formation of chloroplasts and the accumulation of chlorophyll in plants (Xu et al., 2012). In this study, N application significantly increased the chlorophyll a and total chlorophyll content of bermudagrass (Figures 6A,B and Supplementary Table 1). However, the chlorophyll content could not increase in proportion when N was applied over 10 mM, which was consistent with previous studies (Zhang et al., 2013; Yang et al., 2015; Shao et al., 2020). N deficiencies can also decrease leaf area and intensity of photosynthesis (Zhang et al., 2013). Previous studies reported that N application significantly improved physiological parameters and elevated the photosynthetic capacity of leaves in some plant species (Siddiqui et al., 2010; Zhang et al., 2013). However, other studies showed that the promotion of N application on photosynthetic characteristics might also have concentration effects. For instance, N fertilizer can improve the photosynthetic characteristics of soybeans and ryegrass, but their effects were gradually inhibited or decreased with the increase in N fertilization application level (Zhang et al., 2013; Shao et al., 2020). In our study, the chlorophyll fluorescence intensity of bermudagrass leaves grown under moderate N (10 mM) was highest compared with 15 or 2 mM N, especially under high salt conditions (Figure 6). Under salt stress, excessive application of N could not promote but weaken the fluorescent transients of bermudagrass, especially the J (2 ms) and P (30 ms) steps, suggesting that N over application under salt stress might lead to the photosynthetic electron transport traffic jam, especially beyond Q_A_^–^ (Figure 6). Moreover, the optimum amount of N might promote primary photochemical reactions of PSII under high salt stress. The PI_ABS_ and PICS value, which could accurately reflect the state of plant photosynthetic apparatus, peaked under 10 mM N (Figure 6E), indicating that N could promote the primary photochemical reactions of PSII in the waterside. Changes in various quantum efficiencies per reaction center reflected the elevated absorption and transformation of light energy and the reduction in heat dissipation of leaves after appropriate N application (Figures 6E,F; Yusuf et al., 2010).

For forage bermudagrass, the yield and forage quality maintenance should be taken into consideration when evaluating the optimal N application under salt stress. We noticed that different N application levels had no significantly different effects on biomass under NaCl treatment, which is consistent with the previous study in other plants (Jia et al., 2017). However, the plant height had a lower value when plants were grown with 10 mM N compared with 15 mM N. The reason might be because those effectors contributing to the biomass, such as the decrease in firing rate (Figure 1D), might make up for the possible biomass reduction. Nitrogen application has critical effects on forage quality traits (e.g., crude protein, crude fat, and crude fiber) (Collins, 1991). In this study, under salt stress, when 10 mM N was added, the crude protein of green leaves had the highest value compared with 15 mM N (Figure 4D). The similar tendency of N content and crude protein content suggested that N might be redistributed among different tissues under salt stress and more N might be transferred into the green leaves to synthesize crude protein after being supplied with moderate N in bermudagrass. The constituents of crude fiber and crude cash are both the most remarkable factors to evaluate forage quality, which can limit forage intake and digestibility (Bruun et al., 2010; Shaer, 2010; Wolf et al., 2012). In this study, 10 mM N application could maintain crude fiber content at a lower level with the lowest crude ash content under high salt conditions, suggesting better maintenance of forage quality when supplied with moderate N.

Together with the previous studies, N requirements for plants in a saline environment might be inconsistent with those in the normal environment and salt stress might lead to the reduction in nutrients absorbed from the surrounding environment (Shenker et al., 2003; Ramos et al., 2012; Zhang et al., 2012). For forage bermudagrass, the optimal N application under salt stress should be considered in terms of plant growth, salt-stress-responsive physiological parameters, and nutrient value maintenance. Moderate reduction in N application had the optimal effect on alleviating the damage of high salt stress to forage bermudagrass through regulating numerous interconnected physico-chemical activities. These results obtained in this study are important for the agricultural practice to guide rational N fertilization application rather than in excess on bermudagrass, especially under severe salinity environment to reduce N leaching and environmental burden.

## Data Availability Statement

The original contributions presented in the study are included in the article/Supplementary Material, further inquiries can be directed to the corresponding author/s.

## Author Contributions

AS and HW performed the experimental design and data analysis. AS wrote the first draft of the manuscript. XX and XL performed the experiments. EA modified the manuscript draft. JF supervised all the experiments. All authors contributed to seen and the study conception and design and approved the manuscript.

## Conflict of Interest

The authors declare that the research was conducted in the absence of any commercial or financial relationships that could be construed as a potential conflict of interest.

## Publisher’s Note

All claims expressed in this article are solely those of the authors and do not necessarily represent those of their affiliated organizations, or those of the publisher, the editors and the reviewers. Any product that may be evaluated in this article, or claim that may be made by its manufacturer, is not guaranteed or endorsed by the publisher.
