# Supplementary material for: Moderately Reducing Nitrogen Application Ameliorates Salt-Induced Growth and Physiological Damage on Forage Bermudagrass
Source: Front Plant Sci. 2022 Apr 29;13:896358. doi: 10.3389/fpls.2022.896358 (PMC9100817; doi:10.3389/fpls.2022.896358)
Supplement: Supplementary file 1 [file Data_Sheet_1.PDF]

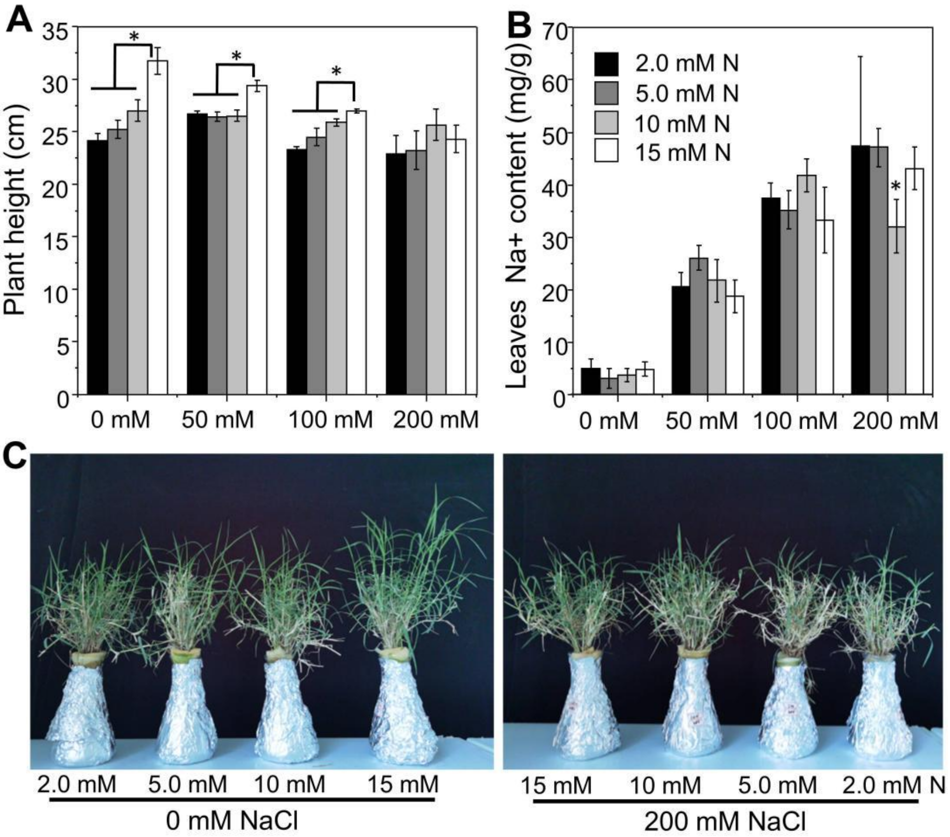


**Supplementary Figure 1.** The growth of bermudagrass grown under different treatment. The plant height (A), $Na^+$ content (B) in the leaves of bermudagrass supplied with different N levels (2.0, 5.0, 10, 15 mM) grown under different NaCl levels (0, 50, 100, 200 mM). C, Image of plants supplied with different N concentration grown under different NaCl level. Different letters above the columns indicate statistically significant differences at $P < 0.05$ under different N levels with the same NaCl level by Tukey's test. * showed significant difference ($P < 0.05$) under different N concentration with the same NaCl level by Tukey's test.

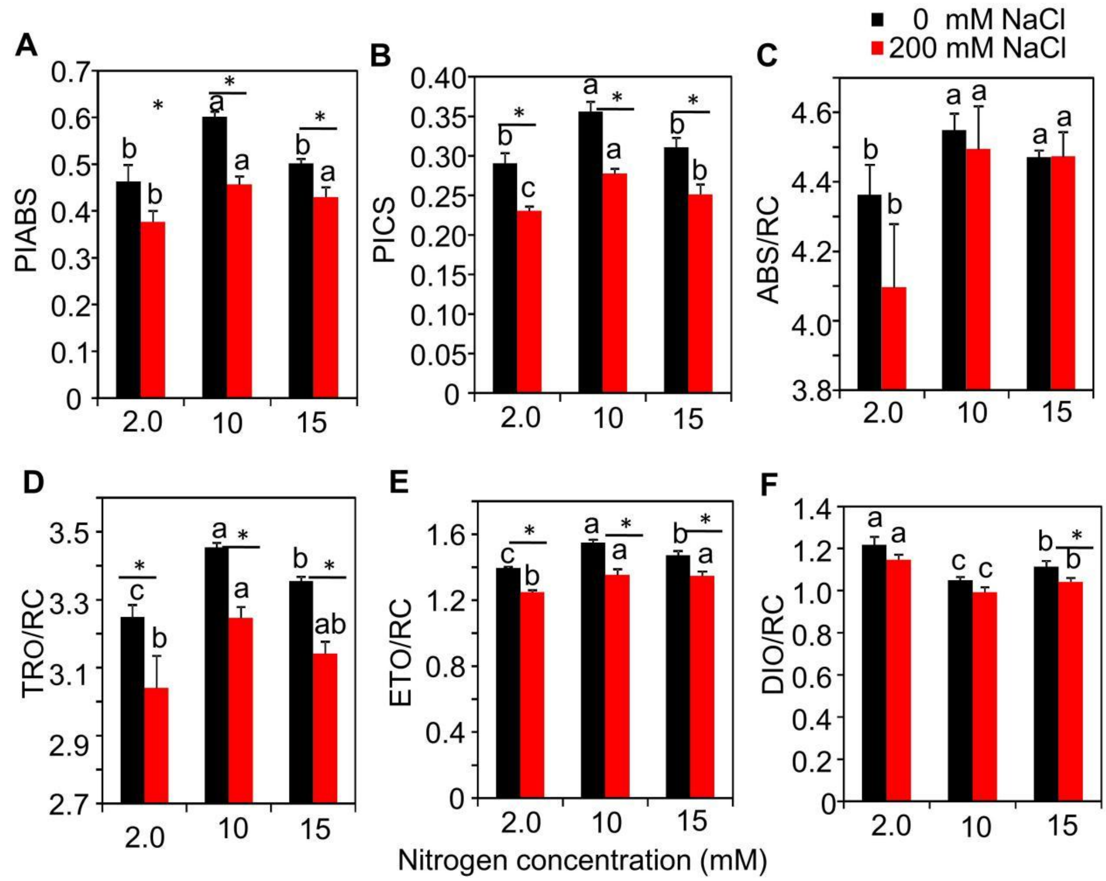


**Supplemental Figure 2.** The Chlorophyll fluorescence parameters of bermudagrass under different treatment. $PI_{ABS}$ (A), $PI_{CS}$ (B), ABS/RC (C), TRo/RC (D), ETo/RC (E), DIo/RC (F) of leaves grown with different nitrogen concentrations exposed to different NaCl level respectively. Different letters above the columns indicate statistically significant differences at $P < 0.05$ under different N levels with the same NaCl level by Tukey's test. * showed significant differences ($P < 0.05$) under 200 mM NaCl treatment and 0 mM NaCl condition with the same N level by Tukey's test.

**Supplemental Table 1** Results of two-way analyses of variance (ANOVA) for analyses of difference in growth related trait to examine the effects of "N", "Salt" and "N*Salt".

| Variable | factor | df | F | *P* |
|---|---|---|---|---|
| | N | 2 | 42.1 | 0 |
| Plant Height | Salt | 1 | 2402.5 | 0 |
| | N * Salt | 2 | 51.1 | 0 |
| | N | 2 | 3.468 | 0.065 |
| Biomass | Salt | 1 | 34.766 | 0 |
| | N * Salt | 2 | 3.953 | 0.048 |
| | N | 2 | 46.352 | 0 |
| Chlorophyll a | Salt | 1 | 0.222 | 0.646 |
| | N * Salt | 2 | 0.495 | 0.621 |
| | N | 2 | 5.644 | 0.019 |
| Chlorophyll | Salt | 1 | 5.667 | 0.035 |
| | N * Salt | 2 | 0.115 | 0.892 |

**Supplemental Table 2** Two-way ANOVA for analyses of difference in $Na^+$ and $Na^+/K^+$ to examine the effects of "N", "Salt" and "N*Salt".

| Variable | factor | df | F | *P* | Variable | factor | df | F | *P* |
|---|---|---|---|---|---|---|---|---|---|
| | N | 2 | 26.588 | 0 | | N | 2 | 6.654 | 0.011 |
| GL $Na^+$ | Salt | 1 | 1576.088 | 0 | GL $Na^+/K^+$ | Salt | 1 | 1444.991 | 0 |
| | N * Salt | 2 | 23.547 | 0 | | N * Salt | 2 | 8.957 | 0.004 |
| | N | 2 | 71.725 | 0 | | N | 2 | 0.133 | 0.877 |
| S $Na^+$ | Salt | 1 | 1820.888 | 0 | S $Na^+/K^+$ | Salt | 1 | 461.519 | 0 |
| | N * Salt | 2 | 48.256 | 0 | | N * Salt | 2 | 3.187 | 0.078 |
| | N | 2 | 136.616 | 0 | | N | 2 | 4.649 | 0.032 |
| OL $Na^+$ | Salt | 1 | 813.127 | 0 | OL $Na^+/K^+$ | Salt | 1 | 279.317 | 0 |
| | N * Salt | 2 | 35.607 | 0 | | N * Salt | 2 | 3.275 | 0.073 |
| | N | 2 | 2.518 | 0.122 | | N | 2 | 1.671 | 0.229 |
| R $Na^+$ | Salt | 1 | 3308.065 | 0 | R $Na^+/K^+$ | Salt | 1 | 3173.621 | 0 |
| | N * Salt | 2 | 1.374 | 0.29 | | N * Salt | 2 | 1.049 | 0.38 |

Green leaves (GL), stems (S), old leaves (OL) and roots (R)

**Supplemental Table 3** Two-way ANOVA for analyses of difference in nitrogen and crude protein content to examine the effects of “N”, “Salt” and “N*Salt”.

| Variable | factor | df | F | *P* | Variable | factor | df | F | *P* |
|---|---|---|---|---|---|---|---|---|---|
| | N | 2 | 6.654 | 0.011 | | N | 2 | 32.822 | 0 |
| GL N content | Salt | 1 | 156.433 | 0 | GL crude protein | Salt | 1 | 157.992 | 0 |
| | N * Salt | 2 | 5.685 | 0.018 | | N * Salt | 2 | 7.919 | 0.006 |
| | N | 2 | 24.17 | 0 | | N | 2 | 23.404 | 0 |
| S N content | Salt | 1 | 13.22 | 0.003 | S crude protein | Salt | 1 | 14.922 | 0.002 |
| | N * Salt | 2 | 71.014 | 0 | | N * Salt | 2 | 69.66 | 0 |
| | N | 2 | 0.403 | 0.677 | | N | 2 | 2.946 | 0.091 |
| OL N content | Salt | 1 | 53.804 | 0 | OL crude protein | Salt | 1 | 61.544 | 0 |
| | N * Salt | 2 | 12.857 | 0.001 | | N * Salt | 2 | 14.839 | 0.001 |

Green leaves (GL), stems (S) and old leaves (OL).

**Supplemental Table 4** Results of ANOVAs for analyses of difference in gene expression to examine the effects of “N”, “Salt” and “N*Salt”.

| Variable | factor | df | F | *P* |
|---|---|---|---|---|
| | N | 2 | 13.737 | 0.001 |
| *NR* expression | Salt | 1 | 10.204 | 0.008 |
| | N * Salt | 2 | 4.426 | 0.036 |
| | N | 2 | 8.47 | 0.005 |
| *AMT* expression | Salt | 1 | 3.271 | 0.096 |
| | N * Salt | 2 | 4.831 | 0.029 |
| | N | 2 | 1.574 | 0.247 |
| *GS* expression | Salt | 1 | 0.134 | 0.721 |
| | N * Salt | 2 | 16.275 | 0 |
| | N | 2 | 13.21 | 0.001 |
| *GOGAT* expression | Salt | 1 | 0.137 | 0.717 |
| | N * Salt | 2 | 23.706 | 0 |

**Supplemental Table 5** Two-way ANOVA for analyses of difference in quality-related traits to examine the effects of "N", "Salt" and "N*Salt".

| Variable | factor | df | F | *P* |
|---|---|---|---|---|
| crude fat | N | 2 | 1.123 | 0.357 |
| | Salt | 1 | 37.747 | 0 |
| | N * Salt | 2 | 7.667 | 0.007 |
| crude fiber | N | 2 | 13.626 | 0.001 |
| | Salt | 1 | 108.15 | 0 |
| | N * Salt | 2 | 56.469 | 0 |
| crude ash | N | 2 | 5.532 | 0.02 |
| | Salt | 1 | 72.107 | 0 |
| | N * Salt | 2 | 4.179 | 0.042 |

**Supplemental Table 6** Primers for gene expression analysis of nitrogen metabolism-related genes.

| Gene | Forward primer | Reverse primer |
|---|---|---|
| *CdACT* | TCTGAAGGGTAAGTAGAGTAG | ACTCAGCACATTCCAGCAGAT |
| *NR* | AGATCGGTCTGGTGTTCGAG | GCACCTCTGACATGGTGAAC |
| *AMT* | AGACGTCGATCACGTTCCAG | GCTCCTTCAACACCATCCTC |
| *GS* | TACAGCACAAAGACCATGCG | AGCTGTCTATGCTAGCCGTT |
| *GOGAT* | TGAAGGCCCTTGAGGTAGTG | TCGCTTCAGGACCAAGGAAT |
